# Association between non-high-density lipoprotein cholesterol to high-density lipoprotein cholesterol ratio (NHHR) and urinary albumin-creatinine ratio (ACR) in US adults: NHANES 2005–2018

**DOI:** 10.1371/journal.pone.0325843

**Published:** 2025-06-10

**Authors:** Chaoying Yong, Hongbin He, Chaohui Zhou, Xiaoqin Zhang, Tian Li, Juan Dong, Yingjuan Zhang

**Affiliations:** Department of Nephrology, Panzhihua Central Hospital, Panzhihua, China; Istituto Di Ricerche Farmacologiche Mario Negri, ITALY

## Abstract

**Purpose:**

The ratio of non-high-density lipoprotein cholesterol (NHDL-C) to high-density lipoprotein cholesterol (HDL-C), referred to as NHHR, is an emerging lipid parameter. The relationship between NHHR and the urinary albumin-creatinine ratio (ACR) remains unclear. Therefore, our aim is to explore the potential correlation between NHHR and ACR.

**Methods:**

Data for this study were sourced from the 2005–2018 National Health and Nutrition Examination Survey (NHANES). Participants under 20 years, pregnant individuals, and those missing NHHR or ACR data were excluded. NHHR, calculated as the difference between total cholesterol (TC) and HDL-C divided by HDL-C, was assessed. Our analysis of the NHHR-ACR association involved multivariable linear regression, smoothed curve fitting, and subgroup analysis.

**Results:**

A total of 34,734 participants were included, with a mean age of 49.81 ± 17.66 years. The prevalence of albuminuria was 12.66%. Multivariable regression analysis indicated a significant and independent positive association between NHHR and ACR after fully adjustment [β (95% CI): 7.19 (4.54, 9.85), p < 0.0001], particularly among female participants, individuals aged 60 or older, those of Mexican American or Non-Hispanic Black ethnicity, individuals with obesity, hypertension or diabetes.

**Conclusion:**

Our findings demonstrate a positive correlation between NHHR and elevated ACR in US adults, implying that lowering NHHR may serve as a preventive and palliative strategy for albuminuria.

## Introduction

Chronic kidney disease (CKD), characterized by its chronic and progressive nature, poses a substantial threat to global public health due to its significant morbidity and mortality rates [1,2]. According to the Global Burden of Disease Study, CKD ranked as the 16th leading cause of death worldwide in 2016, climbing to the 10th position by 2019. Projections indicate that by 2040, CKD is anticipated to become the 5th leading cause of death globally [3]. The urinary albumin creatinine ratio (ACR) serves as a reliable marker for assessing glomerular injury and urinary albumin levels. Defined as an ACR ≥ 30 mg/g, albuminuria represents a crucial diagnostic indicator during the initial phases of CKD and is linked with an adverse prognosis [4,5]. Multiple studies have shown that albuminuria independently predicts cardiovascular risk, resulting in a considerable increase in both all-cause mortality and cardiovascular mortality, even when ACR levels are below 30 mg/g [6–8]. Consequently, ACR has been incorporated into the risk stratification and management protocols for both chronic kidney disease and cardiovascular disease [9,10].

The pathogenesis of CKD remains elusive, with a multitude of identified risk factors. Among these, hyperlipidemia has received increasing attention [11]. Numerous studies have demonstrated that dyslipidemia exacerbates the progression of CKD [12–14]. Dyslipidemia induces oxidative stress, mitochondrial dysfunction, lipotoxicity, and insulin resistance, thereby augmenting the risk of albuminuria [15,16]. As a novel lipid complex parameter, the NHHR has been demonstrated in previous studies to exhibit good predictive value in a variety of diseases, including atherosclerosis, cerebrovascular disease, non-alcoholic fatty liver disease, kidney stones, and metabolic syndrome [17–20]. However, research exploring the relationship between NHHR and ACR remains limited.

Hence, this study aims to clarify the association between NHHR and ACR utilizing data from the NHANES spanning from 2005 to 2018.

## Materials and methods

### Survey description

The NHANES, overseen by the National Center for Health Statistics (NCHS), conducts surveys to gather demographic information on the health and nutritional habits of U.S. residents. This comprehensive database encompasses demographic details, dietary patterns, health status, physical examinations, and laboratory test results. All NHANES study protocols received approval from the Research Ethics Review Board of NCHS, and written informed consent was obtained from all participants. The required data was sourced from the official NHANES website (https://www.cdc.gov/nchs/nhanes/).

### Study population

**Fig 1:**
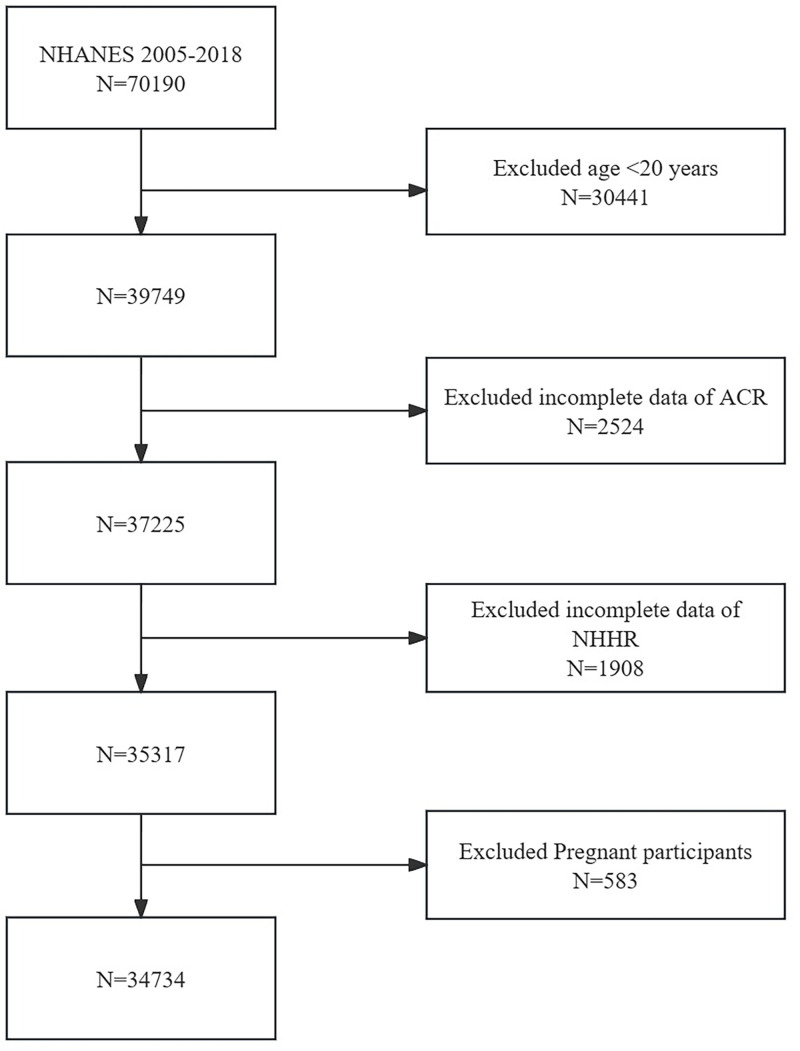
Flowchart of the study design and participants.

We enlisted data from seven NHANES cycles spanning from 2005 to 2018 to evaluate the relationship between NHHR and ACR. Initially, 70,190 participants were enrolled in the study. However, after applying exclusion criteria: (1) age < 20 years old (n = 30,441), (2) incomplete data regarding ACR (n = 2,524) and NHHR (n = 1,908), and (3) pregnancy (n = 583), a total of 34,734 eligible subjects remained for our final analysis (Fig 1).

### Definition of NHHR

Enzymatic assays, conducted with an automated biochemical analyzer, were employed to evaluate TC and HDL-C levels in fasting individuals. The primary exposure factor investigated in this study was NHHR, defined as the ratio of NHDL-C to HDL-C. NHDL-C is calculated as TC minus HDL-C.

### Definition of ACR

ACR was calculated by dividing the urinary albumin concentration (mg) by the urinary creatinine concentration (g). Urinary albumin and creatinine levels were assessed using a solid-phase fluorescence immunoassay and modified Jaffe kinetics, respectively, using a single-spot urine sample. Albuminuria is defined as a urinary ACR exceeding 30 mg/g. During statistical analyses, ACR was treated as a continuous outcome variable.

### Covariates

Our study encompassed various covariates potentially influencing the association between NHHR and ACR. These included age (year), gender (male/female), race (Mexican American/other Hispanic/non-Hispanic White/non-Hispanic Black/other races), education level (less than high school/high school or general educational development/above high school/others), marital status (married/single/with partner), family poverty income ratio (PIR), body mass index (BMI, kg/m²), waist circumference (cm), smoking status (yes/no), alcohol consumption (yes/no/unknown), hypertension (yes/no), diabetes (yes/no), AST (U/L), ALT (U/L), total protein (g/L), albumin (g/L), serum uric acid (μmol/L), serum urea nitrogen (mg/dl), and serum creatinine (μmol/L). BMI categories were defined as <18.5, ≥ 18.5 to <25, ≥ 25 to <30, and ≥30 kg/m², representing underweight, normal weight, overweight, and obese individuals, respectively. Diabetes was defined as the use of hypoglycemic drugs, insulin injections, a physician’s diagnosis of diabetes, a hemoglobin A1c level of ≥6.5%, fasting blood glucose ≥7.0 mmol/L, or a 2-hour plasma glucose ≥11.1 mmol/L. Hypertension was defined as the use of antihypertensive medication, a physician’s diagnosis of hypertension, or three consecutive systolic blood pressure measurements ≥140 mmHg or diastolic blood pressure ≥90 mmHg [21]. Detailed measurement procedures for these variables are available at the following link: https://www.cdc.gov/nchs/nhanes/.

### Statistical analysis

All statistical analyses were conducted in accordance with CDC guidelines. Categorical data were described using frequencies and percentages, while continuous variables were presented as means ± standard deviations(SD). Differences among participants grouped by NHHR quartiles were evaluated using either a weighted Student’s t-test (for continuous variables) or a weighted chi-square test (for categorical variables). Multivariable linear regression models were utilized to examine the relationship between NHHR and ACR. Model 2 was adjusted for sex, age, and race, while model 3 additionally incorporated adjustments for education, marital status, body mass index, waist circumference, smoking, alcohol consumption, hypertension, diabetes mellitus, glutamic oxaloacetic transaminase, glutamic alanine aminotransferase, total protein, albumin, serum uric acid, serum urea nitrogen, and serum creatinine. Smoothed curve fitting was applied to address the nonlinearity of NHHR with ACR. Additionally, subgroup analysis and interaction analyses were conducted to explore potential differences between various populations. Missing values were imputed using the median for continuous variables or mode for categorical variables based on existing cases. All analyses were performed using Empower software (www.empowerstats.com). A significance level of p < 0.05 was adopted.

## Results

### Baseline characteristics of the study population

A total of 34,734 participants were selected from NHANES 2005–2018, with a gender distribution of 49.27% males and 50.73% females. The average age was 49.81 ± 17.66 years. Quartile ranges for NHHR were delineated as 0.21–1.92, 1.93–2.66, 2.67–3.64, and 3.65–26.85. Albuminuria prevalence was 12.66%. Individuals in the Q4 group exhibited higher ACR compared to the Q1 group (62.48 ± 450.42 vs. 42.54 ± 319.26). Statistical differences across the four NHHR quartiles were observed in various demographic and clinical parameters, including age, gender, race, education level, marital status, family poverty income ratio, BMI, waist circumference, smoking status, alcohol consumption, hypertension, diabetes, aspartate transaminase (AST), alanine transaminase (ALT), total protein, albumin, serum uric acid, serum urea nitrogen, serum creatinine, TC, HDL-C, urinary albumin, urinary creatinine, and ACR (Table 1).

**Table 1:** Baseline characteristics of the study population according to NHHR quartiles(n = 34734).

Characteristic	Total	Q1 (0.21–1.92)	Q2 (1.93–2.66)	Q3 (2.67–3.64)	Q4 (3.65–26.85)	P-value
N	34734	8569	8736	8713	8716	
Age (year)	49.81 ± 17.66	49.53 ± 19.39	50.45 ± 18.38	50.22 ± 17.03	49.02 ± 15.61	<0.001
Gender(%)						<0.001
Male	49.27	35.70	43.30	53.28	64.59	
Female	50.73	64.30	56.70	46.72	35.41	
Race(%)						<0.001
Mexican American	15.84	11.11	14.14	17.86	20.17	
Other Hispanic	9.72	7.33	9.41	10.55	11.54	
Non-Hispanic White	42.34)	42.37	42.11	41.55	43.35	
Non-Hispanic Black	20.78	27.24	22.84	19.17	13.96	
Other Race	11.33	11.95	11.50	10.88	10.98	
Education level(%)						<0.001
Less than high school	24.92	20.47	23.08	26.09	29.97	
High school or GED	22.84	21.23	22.44	23.95	23.72	
Above high school	52.15	58.17	54.44	49.91	46.18	
Other	0.09	0.13	0.05	0.05	0.14	
Marital status(%)						<0.001
Married	52.00	45.66	50.66	54.93	56.63	
Single	39.99	46.45	41.55	37.69	34.37	
With partner	8.01	7.89	7.78	7.38	8.99	
Family PIR	2.48 ± 1.56	2.60 ± 1.59	2.53 ± 1.57	2.46 ± 1.54	2.34 ± 1.52	<0.001
BMI(kg/m2)	29.17 ± 6.88	26.48 ± 6.53	28.84 ± 6.98	30.21 ± 6.77	31.11 ± 6.30	<0.001
Waist Circumference (cm)	99.28 ± 15.96	91.74 ± 15.27	98.01 ± 15.72	102.10 ± 15.23	105.12 ± 14.38	<0.001
Smoke(%)						<0.001
Yes	44.74	41.51	42.23	44.65	50.53	
No	55.26	58.49	57.77	55.35	49.47	
Alcohol(%)						<0.001
Yes	61.94	65.05	61.00	60.81	60.95	
No	17.33	14.13	17.06	18.03	17.33	
Unknown	20.73	20.82	21.94	21.16	19.01	
Hypertension(%)						<0.001
Yes	37.78	35.86	35.93	38.51	40.78	
No	62.22	64.14	64.07	61.49	59.22	
Diabetes(%)						<0.001
Yes	18.80	16.19	17.41	19.38	22.19	
No	81.20	83.81	82.59	80.62	77.81	
AST (U/L)	25.48 ± 16.85	25.31 ± 18.86	24.47 ± 15.87	25.13 ± 16.07	27.00 ± 16.34	<0.001
ALT (U/L)	25.13 ± 20.37	21.49 ± 17.89	22.88 ± 20.58	25.47 ± 19.82	30.61 ± 21.77	<0.001
Total protein (g/L)	71.60 ± 4.66	71.15 ± 4.74	71.35 ± 4.64	71.77 ± 4.55	72.13 ± 4.64	<0.001
Albumin (g/L)	42.27 ± 3.39	42.26 ± 3.49	42.11 ± 3.43	42.21 ± 3.34	42.51 ± 3.27	<0.001
Serum uric acid (μmol/L)	325.23 ± 85.74	296.63 ± 80.79	314.70 ± 82.13	334.27 ± 83.83	354.87 ± 84.98	<0.001
Serum urea nitrogen (mg/dl)	13.75 ± 5.96	13.64 ± 6.36	13.76 ± 5.86	13.86 ± 6.00	13.73 ± 5.60	<0.001
Serum creatinine (μmol/L)	79.81 ± 33.69	78.21 ± 38.90	78.68 ± 27.94	80.33 ± 32.84	82.02 ± 34.15	<0.001
TC(mmol/L)	4.99 ± 1.08	4.40 ± 0.90	4.73 ± 0.89	5.06 ± 0.91	5.75 ± 1.10	<0.001
HDL-C(mmol/L)	1.37 ± 0.42	1.80 ± 0.43	1.44 ± 0.28	1.23 ± 0.23	1.01 ± 0.20	<0.001
Albumin, urine (mg/L)	48.02 ± 345.70	38.43 ± 233.77	41.31 ± 290.97	47.64 ± 352.08	64.54 ± 461.94	<0.001
Creatinine, urine (mg/dL)	124.40 ± 80.64	115.48 ± 79.64	122.42 ± 81.76	126.37 ± 79.70	133.20 ± 80.42	<0.001
ACR(mg/g)	48.15 ± 363.39	42.54 ± 319.26	40.89 ± 283.50	46.63 ± 377.37	62.48 ± 450.42	<0.001
Albuminuria(%)						<0.001
Yes	12.66	12.47	11.81	12.41	13.94	
No	87.34	87.52	88.19	87.59	86.06	

Q1–Q4: Grouped by quartile according to the NHHR. Continuous variables are presented as mean ± standard deviation (SD), while categorical variables are expressed as percentages.

PIR, poverty income ratio; BMI, body mass index; ALT, alanine transaminase; AST, aspartate transaminase; TC, total cholesterol; HDL-C, high-density lipoprotein-cholesterol; ACR, urine albumin-to-creatinine ratio.

### The association between NHHR and ACR

Our findings demonstrated a positive association between NHHR and ACR (Table 2). This relationship remained significant across different models, including the crude model [β (95% CI): 7.08 (4.45, 9.70), p < 0.0001], the minimally adjusted model [β (95% CI): 8.09 (5.38, 10.80), p < 0.0001], and the fully adjusted model [β (95% CI): 7.19 (4.54, 9.85), p < 0.0001]. In the fully adjusted model, each unit increase in NHHR was associated with a 7.19 mg/g increased in ACR. Furthermore, NHHR was categorized into quartiles, and the positive correlation with ACR persisted in both linear and categorical analyses. Compared to the lowest NHHR quartile, participants in the highest quartile exhibited a significantly higher ACR [β (95% CI): 22.39 (11.39, 33.40), p < 0.0001].

**Table 2:** Association between NHHR and ACR.

Outcome	β (95%CI)^1^, p-Value		
	Crude model(Model 1)^2^	Minimally adjusted model(Model 2)^3^	Fully adjusted model(Model 3)^4^
NHHR (continuous)	7.08 (4.45, 9.70) <0.0001	8.09 (5.38, 10.80) <0.0001	7.19 (4.54, 9.85) <0.0001
NHHR (quartiles)			
Q1	Reference	Reference	Reference
Q2	−1.65 (−12.48, 9.18) 0.7651	−2.57 (−13.40, 8.27) 0.6425	2.64 (−7.47, 12.74) 0.6092
Q3	4.09 (−6.75, 14.92) 0.4595	3.64 (−7.31, 14.58) 0.5152	7.19 (−3.27, 17.65) 0.1781
Q4	19.93 (9.10, 30.77) 0.0003	22.12 (10.97, 33.28) 0.0001	22.39 (11.39, 33.40) <0.0001
p for trend	<0.0001	<0.0001	<0.0001

^1^95% Cl: 95% confidence interval.

^2^Crude Model: no covariates were adjusted.

^3^Minimally adjusted model: adjusted for age, gender, and race.

^4^Fully adjusted model: adjusted for age, gender, race, education level, marital status, BMI, waist Circumference, smoke, alcohol, hypertension, diabetes, AST, ALT, total protein, albumin, serum uric acid, serum urea nitrogen, serum creatinine.

In the fully adjusted model, several variables were significantly associated with increased ACR (Table 3). Specifically, ACR increased by 1.25 mg/g for each year of age (p < 0.0001). Female participants exhibited a higher ACR of 45.16 mg/g compared to male participants (p < 0.0001). Furthermore, compared to Mexican Americans, other Hispanics (p = 0.0171), non-Hispanic Whites (p < 0.0001), and non-Hispanic Blacks (p < 0.0001) exhibited reduced ACRs of 17.49 mg/g, 40.61 mg/g, and 75.38 mg/g, respectively. Moreover, for each unit increase in family PIR, ACR decreased by 4.34 mg/g (p = 0.0010). Conversely, for each unit increase in body mass index (BMI), ACR increased by 1.32 mg/g (p < 0.0001). Furthermore, ACR increased by 47.59 mg/g and 89.54 mg/g in individuals with hypertension and diabetes, respectively, compared to their counterparts (all p < 0.0001). Additionally, for each unit increase in albumin and serum uric acid, ACR decreased by 16.46 mg/g and 0.13 mg/g, respectively (all p < 0.0001). Contrarily, for each unit increase in serum urea nitrogen, serum creatinine, and TC, ACR was elevated by 2.64 mg/g, 3.86 mg/g, and 23.66 mg/g, respectively (all p < 0.0001).

**Table 3:** Multivariable linear regression models of ACR.

Variables	β (95%CI)1	p-Value
NHHR	7.19 (4.54, 9.85)	<0.0001
Age (year)	1.25 (1.03, 1.46)	<0.0001
Female (versus male)	45.16 (36.00, 54.33)	<0.0001
Race (versus Mexican American)		
Other Hispanic	−17.49 (−31.87, −3.12)	0.0171
Non-Hispanic White	−40.61 (−52.01, −29.21)	<0.0001
Non-Hispanic Black	−75.38 (−88.14, −62.62)	<0.0001
Other Race	−11.06 (−25.46, 3.35)	0.1324
Education level (versus Less than high school)		
High school or GED	−5.48 (−16.04, 5.08)	0.3095
Above high school	−6.42 (−16.28, 3.43)	0.2016
Other	−44.85 (−166.12, 76.42)	0.4686
Marital status (versus married)		
Single	−1.73 (−9.51, 6.05)	0.6626
With partner	−5.10 (−18.92, 8.72)	0.4697
Family PIR	−4.34 (−6.93, −1.76)	0.0010
BMI	1.32 (0.76, 1.88)	<0.0001
Waist Circumference	0.37 (−0.14, 0.88)	0.1601
Smoke(yes versus no)	2.29 (−5.32, 9.90)	0.5554
Alcohol(yes versus no)	−5.20 (−15.20, 4.80)	0.3082
Hypertension(yes versus no)	47.59 (39.72, 55.45)	<0.0001
Diabetes(yes versus no)	89.54 (79.61, 99.47)	<0.0001
AST (U/L)	0.04 (−0.29, 0.37)	0.8134
ALT (U/L)	−0.06 (−0.35, 0.22)	0.6636
Total protein (g/L)	0.44 (−0.44, 1.33)	0.3280
Albumin (g/L)	−16.46 (−17.74, −15.17)	<0.0001
Serum uric acid (μmol/L)	−0.13 (−0.18, −0.08)	<0.0001
Serum urea nitrogen (mg/dl)	2.64 (1.86, 3.41)	<0.0001
Serum creatinine (μmol/L)	3.86 (3.73, 4.00)	<0.0001
TC(mmol/L)	23.66 (17.32, 29.99)	<0.0001
HDL-C(mmol/L)	5.78 (−13.40, 24.97)	0.5547

95% CI: 95% confidence interval.

Continuous variables are accompanied by their respective units, while categorical variables include their reference groups. The β coefficient of ACR represents the increase for each unit of the continuous variables and is compared with the reference group for categorical variables.

**Fig 2:**
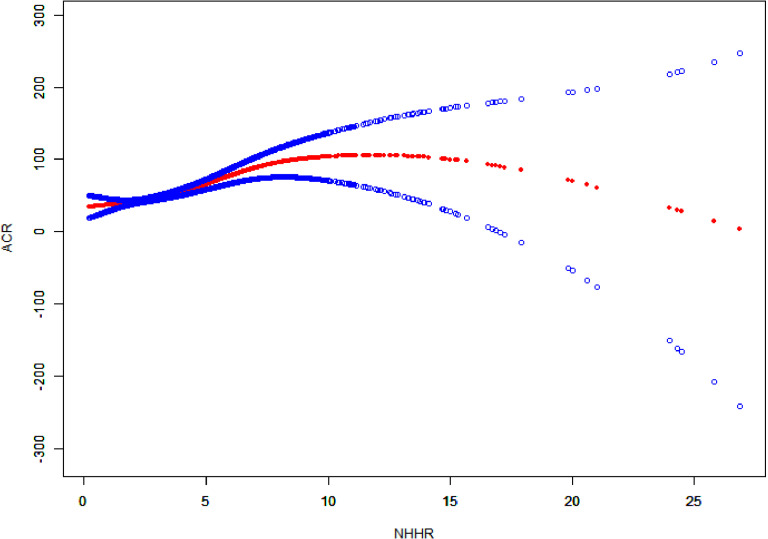
The correlation between NHHR and ACR. The red solid line illustrates the smooth curve fit between the variables, while the blue band represents the 95% confidence interval derived from the fit.

The smooth curve fitting analysis indicated that there was a positive relationship between NHHR and ACR (Fig 2).

### Subgroup analysis

**Fig 3:**
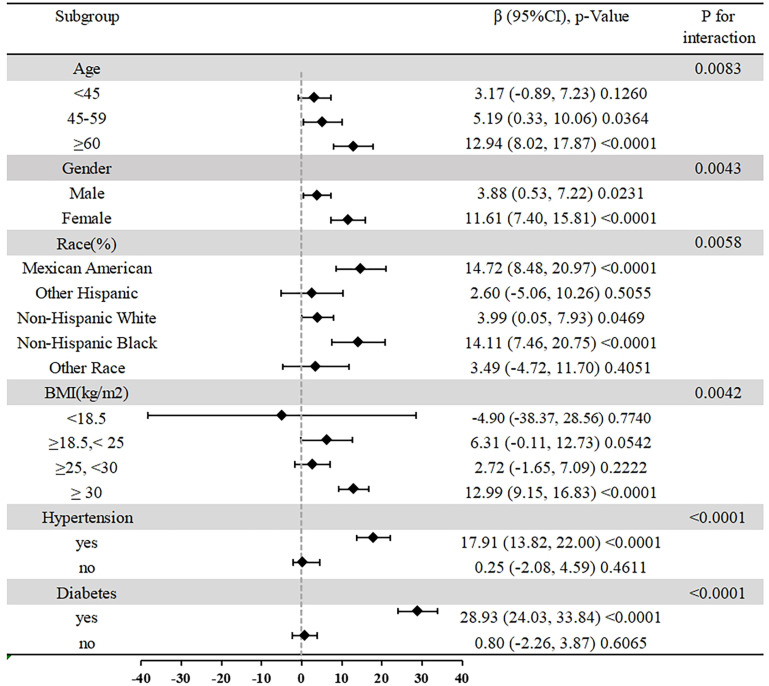
Subgroup analysis for the association between NHHR and ACR.

Subgroup analyses were conducted to assess the stability of the relationship between NHHR and ACR across various demographic groups. The findings revealed a positive association between NHHR and ACR across all subgroups, with greater significance observed in participants who were female, aged ≥ 60, Mexican American, non-Hispanic Black, obese, hypertensive, and diabetic (P < 0.0001). Interaction analyses revealed that age, gender, race, BMI, hypertension, and diabetes influenced the NHHR-ACR association(all P for interaction<0.05) (Fig 3).

## Discussion

This study aimed to examine the relationship between NHHR and ACR. In this cross-sectional analysis of 37,734 participants, we found that ACR levels were higher in individuals with greater NHHR values. Specifically, each unit increase in NHHR was associated with a 7.19 mg/g increase in ACR. Subgroup analyses and interaction tests revealed a positive association between ACR and NHHR across populations, with significant influences from age, sex, race, BMI, hypertension, and diabetes. These findings suggest that reducing NHDL-C levels may serve as a preventive and therapeutic approach for managing albuminuria.

To our knowledge, this is the first study investigating the correlation between NHHR and ACR utilizing the NHANES database. Lipid metabolism disturbances are prevalent in CKD patients and are strongly linked to adverse outcomes [22]. Previous studies have reported associations between various lipid ratio parameters and kidney disease, as lipid ratios appear to better assess the risk of cardiovascular disease compared to individual lipids [23–25]. In a cross-sectional study of a Chinese population, the triglycerides (TG) to HDL-C ratio exhibited a significant positive correlation with UACR, particularly among overweight individuals or those with prediabetes or prehypertension [26]. In a small-sample study of a Greek population, the TG to HDL-C ratio was identified as a predictor of albuminuria in non-diabetic patients [27]. Additionally, in a retrospective analysis, the TC to HDL-C ratio was found to predict the progression of CKD [28]. In contrast to prior research, our study specifically examined the relationship between NHHR and ACR. NHHR incorporates lipid parameters beyond traditional measures, which include very-low-density lipoprotein cholesterol(VLDL-C), intermediate-density lipoprotein (IDL), and apolipoprotein A (apo A), all contributing to atherosclerosis. As a novel lipid indicator, NHHR surpasses conventional lipid parameters in evaluating atherosclerosis risk [29]. Its predictive prowess extends to conditions like NAFLD, metabolic syndrome, and insulin resistance [18,19,30]. The strong correlation of NHHR with diverse diseases underscores its efficacy as a lipid management tool. Our findings affirm a positive correlation between NHHR and ACR, persisting even after adjusting for various covariates. Multivariable regression highlighted significant associations between BMI, hypertension, diabetes mellitus, hyperuricemia, and albuminuria, underscoring the importance of cardiometabolic anomaly awareness in CKD preventive care. Subgroup analyses revealed consistent patterns across various subgroups, reinforcing the robustness of the observed associations. Notably, ACR levels appeared higher in women compared to men, despite similar sample sizes for both genders [21]. This discrepancy may be attributed to lifestyle variations (e.g., smoking, dietary habits), renal structural differences, or hormonal influences [31].

Lipoprotein abnormalities have detrimental effects on kidney function. Prior investigations suggest that familial hyperlipidemia elevates the risk of CKD, indicating an association between systemic lipid levels and CKD [32]. Notably, dyslipidemia often manifests early in CKD progression, marked by decreased HDL and increased low-density lipoproteins (LDL-C) [30]. Statins, the primary class of lipid-lowering medications, have demonstrated efficacy in improving outcomes for CKD patients. A comprehensive meta-analysis of various statins shows a 20% reduction in mortality and major cardiovascular events among CKD patients [33]. The lipid nephrotoxicity hypothesis, proposed by the Moorhead study, suggests that hyperlipidemia triggers inflammation, ROS production, and endogenous oxidative stress, potentially contributing to CKD development [34].

When lipid levels surpass the storage capacity of white adipose tissue, lipids from various sources overflow into non-adipose tissues like the liver, kidneys, pancreas, and muscles, termed ‘ectopic lipid accumulation’ [35]. This phenomenon occurs early in CKD compared to healthy kidneys [36]. Renal lipid accumulation has been linked to glomerulosclerosis. Rats fed a high-fat diet for 32 weeks exhibited chronic inflammation and glomerular fibrosis in their kidneys [37]. Renal biopsies of individuals with obesity-associated nephropathy revealed glomerular hypertrophy and focal segmental glomerulosclerosis(FSGS) lesions [38,39]. Dysregulated renal lipid accumulation is implicated in CD36 overexpression. Studies indicate that mice lacking CD36 exhibit reduced renal lipid accumulation and lower susceptibility to renal injury [40]. In animal trials, CD36 antagonists mitigated renal inflammation and tubulointerstitial fibrosis, thereby slowing CKD progression [41]. Inhibition of CD36 expression holds promise as a therapeutic strategy for addressing chronic renal fibrosis.

Our research has several notable strengths. Utilizing NHANES data, a nationally representative survey adhering to stringent research protocols, our study yields findings with broad applicability to the general U.S. populace. We ensured a robust sample size and accounted for confounding covariates, thus enhancing the study’s reliability. The NHHR, due to its non-invasive and cost-effective nature, presents potential utility in clinical practice for managing and intervening in CKD. However, several important limitations of our study must be acknowledged. Firstly, its cross-sectional design precludes elucidating causal relationships between NHHR and albuminuria. Secondly, while we endeavored to adjust for significant covariates, the influence of other potential confounding variables persists. Finally, the generalizability of our findings to other racial groups is limited by the survey’s focus on the U.S. population.

## Conclusion

To summarize, our study identified a positive correlation between NHHR and ACR. Nevertheless, additional large-scale prospective studies are necessary to conclusively validate our findings.
